# Experimental and numerical investigation of the mechanical properties and energy evolution of sandstone–concrete combined body

**DOI:** 10.1038/s41598-024-53959-4

**Published:** 2024-03-03

**Authors:** Shisong Yuan, Bin Du, Mingxuan Shen

**Affiliations:** College of Civil Engineering, Guizhou University, Huaxi District, Guiyang, 50025 Guizhou China

**Keywords:** Sandstone–concrete combined body (SCCB), Mechanical property, Numerical simulation, Energy, Civil engineering, Environmental impact

## Abstract

Studying the mechanical properties of rock–concrete combined body is crucial to ensure the safety and stability of engineering structures. In this paper, laboratory tests and numerical simulations are used to investigate the mechanical properties of the sandstone–concrete combined body. Uniaxial compression tests and an acoustic emission monitoring system are used to analyze the failure characteristics of the sandstone–concrete sample and to validate the accuracy of the numerical model. The mechanical properties of the composite body were further analyzed by integrating energy and damage theories. The results of the sandstone–concrete study suggest that the combined sandstone–concrete body exhibits synergistic deformation and failure when subjected to uniaxial compression. The peak stress and elastic modulus fall between those of sandstone and concrete. The interface's shape causes the stress in the y-direction to transition from tensile stress to compressive stress. Energy is stored before reaching the peak stress and released after reaching the peak stress. The damage curve indicates that the damage increases gradually with the strain, and it results in plastic failure. In the numerical simulation of triaxial compression, the stress and displacement at the interface are evenly distributed. Compared to uniaxial compression, the energy of each component is higher and shows a linear positive correlation with confining pressure. Additionally, the rate of energy dissipation increases with higher confining pressure. The damage variable also increases with the increase in confining pressure, and the plastic failure process is also apparent under triaxial compression.

## Introduction

Concrete and rock are two of the most commonly used materials in modern engineering. They are important construction materials that provide safety and reliability for engineering projects in road construction, bridge building, tunnel construction, and pile foundation engineering. However, in real-world engineering applications, its mechanical properties are easily influenced by geological vibrations, external loads, the natural environment, and other factors. Therefore, it is essential to study the mechanical properties of rock and concrete to assess the safety of engineering construction and the stability of engineering structures. Furthermore, the current literature review demonstrates that numerous engineering geological disasters occur at the interface between rock and concrete^1,2^.

Both rock and concrete are quasi-brittle materials and their mechanical properties, strength and failure modes are related to cracks, mainly involving crack formation, propagation and joining^3^. Currently, experimental research on rock and concrete materials primarily focuses on complex stress analysis. This involves conducting tests such as uniaxial compression, triaxial compression, step-by-step loading, and cyclic loading and unloading^4^. At the same time, a tensile test is carried out to determine the tensile properties of a single material^5^. In addition, there are studies aimed at predicting material failure from an energy perspective in order to determine the regularity of its occurrence^6^.

Due to ongoing research, the rock-concrete composite, consisting of concrete and rock, has become an important building material that is continually being enhanced and promoted for various applications. Many large-scale hydraulic and transportation projects are constructed directly on rock foundations^7–9^, enabling concrete to interact with the bedrock. This approach is also utilized in tunnel anchors, tunnel support structures, and dams. However, when subjected to an extremely complex external environment and load, the strength, stiffness, and service life of the composite body structure may be diminished, posing a serious threat to the safety and normal operation of engineering structures. Therefore, it is of great engineering significance to study the mechanical properties of the composite material formed by concrete and rock.

Many scholars have carried out a lot of research on rock–concrete combined body. Most of the research on the sandstone–concrete combined body is based on laboratory experimental research, theoretical derivation, numerical simulation and other methods to study the mechanical properties, energy evolution and damage characteristics. To study the interaction of the rock–concrete interface, some scholars have carried out studies on the influence of uniaxial compression, triaxial compression and complex stress on the strength and failure behavior of the SCCB samples^10–12^. From the point of view of high-temperature damage to study the mechanical properties of the SCCB samples, the high temperature on the combined body structure deterioration law^13^. Most of the research on the interfaces of composite materials has focused on the surface roughness and the impact of mechanical parameters on their mechanical properties^14^. These have been investigated through straight shear, tensile, and three-point bending tests^15–20^. There are not many types of research on SCCB materials using numerical software. Most of the numerical software commonly used at present is based on finite element theory. Among them, the numerical software FLAC3D based on the finite difference principle is favored by most scholars. The FLAC3D was used to simulate the force and creep failure of the sample under uniaxial compression and triaxial compression of a single material in the laboratory^21–24^. Many researchers carried out analysis and research under the complex external conditions of freeze–thaw, high temperature and fluid^25,26^. Through the software interface FLAC3D constitutive model secondary development research^27–29^.

Currently, the mechanical properties of the SCCB have become a popular research topic. There are several types of research on the damage and failure characteristics of the SCCB samples In this paper, the powerful geotechnical numerical analysis software FLAC3D is used to develop a numerical model and conduct research on uniaxial compression numerical simulation tests. The study discusses the failure mode and mechanical properties of the sand–concrete combined body under uniaxial compression and numerical conventional triaxial compression, based on the laws of energy evolution and damage evolution.

## Sample preparation and test scheme

### Preparation of sample

This paper studies the SCCB sample, which uses sandstone as the rock material. Sandstone is a sedimentary rock with a high sandy content and is widely distributed throughout the country for easy access. The concrete consists mainly of cement, sand, gravel and water, with a ratio of 1:0.58:0.88:0.51. The cement is ordinary silicate cement PO·42.5.

The obtained cylindrical sandstone samples (height of 100 mm, diameter of 50 mm) were cut into two equal serrated samples with the size of 50 × 50 mm with a linear cutting machine. The serrated samples were 5 mm high and the base length was 12.5 mm. Next, remove the ash layer from the cut rock face. Subsequently, the serrated rock sample was loaded into the prepared mold with a height of 100 mm and a diameter of 50 mm, and the concrete was poured. The concrete was poured over the serrated rock samples to form the sandstone–concrete combined body samples. At the same time, concrete cylinder samples of standard size were poured and uniaxial compression test was carried out.

**Figure 1:**
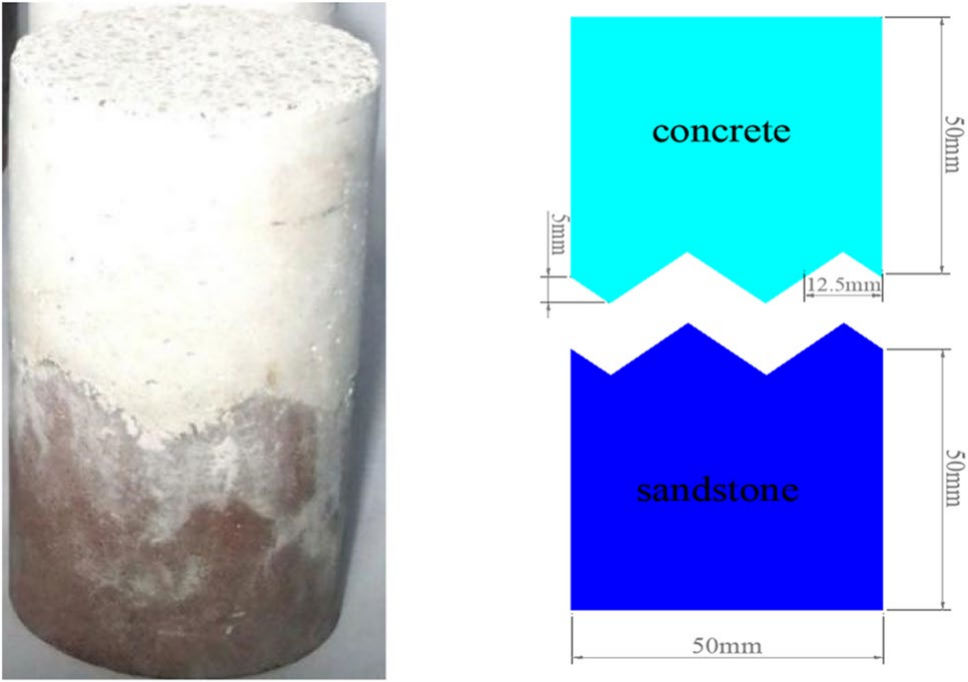
The sandstone–concrete combined body sample.

The sandstone–concrete combined body samples and concrete samples were placed in a curing environment with a temperature of 20 °C and humidity of 96% for curing for 28 days. Finally, all sides of the samples are carefully polished with a grinder until the sample is between 99.7 mm and 100.3 mm in height and 49.7 mm and 50.3 mm in diameter. The shape and size of the prepared cylindrical sandstone–concrete combined body samples are shown in Fig. 1.

### Test equipment and loading scheme

**Figure 2:**
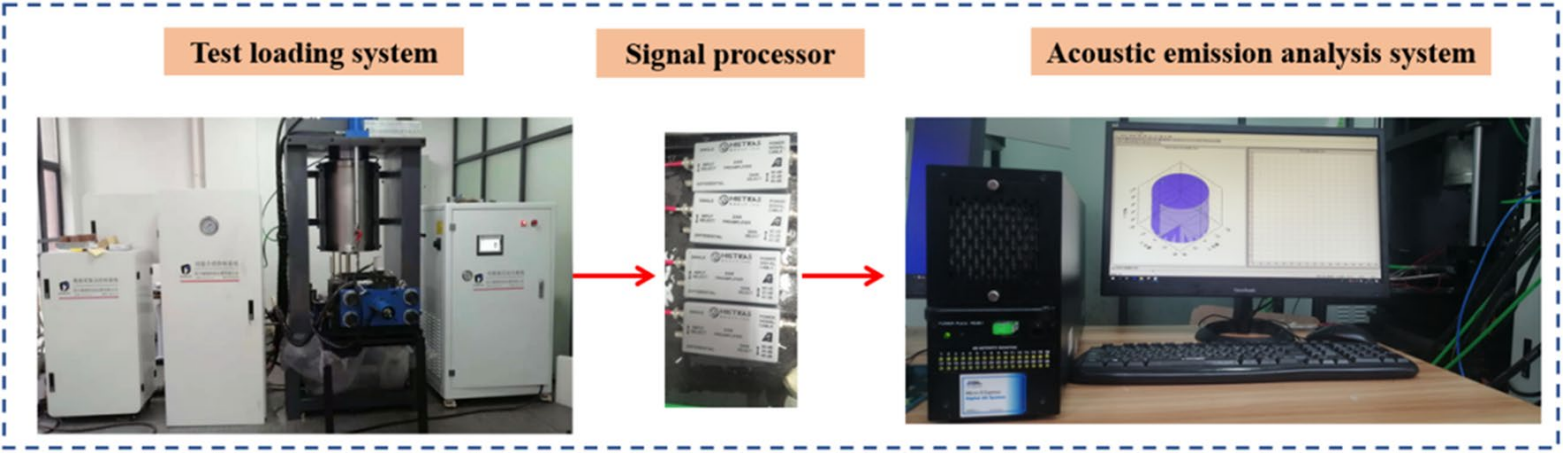
Testing equipment.

The test equipment is shown in Fig. 2, and the servo control material triaxial test system DSZ-1000 was adopted. The technical indicators of this machine are as follows: the maximum load is 1000 KN, the force range is 10–1000 KN. Test force measurement accuracy ≤  ± 0.5%. In this laboratory test, uniaxial compression test was adopted. In the test, the control mode of constant displacement loading was adopted. The loading rate was 0.1 mm/min, and the loading stopped when the sample lost its bearing capacity. The test loading system can automatically collect normal stress and strain and plot stress–strain curves through software operation.

## Analysis of test results

### Analysis of single material test results

In order to make the conclusions on the mechanical properties of the sandstone–concrete combined body more reliable, uniaxial compression tests on a single material of sandstone and concrete are also carried out in this paper. The mechanical properties and failure modes of concrete and sandstone under uniaxial compression were investigated.

#### Failure characteristic analysis of single material

**Figure 3:**
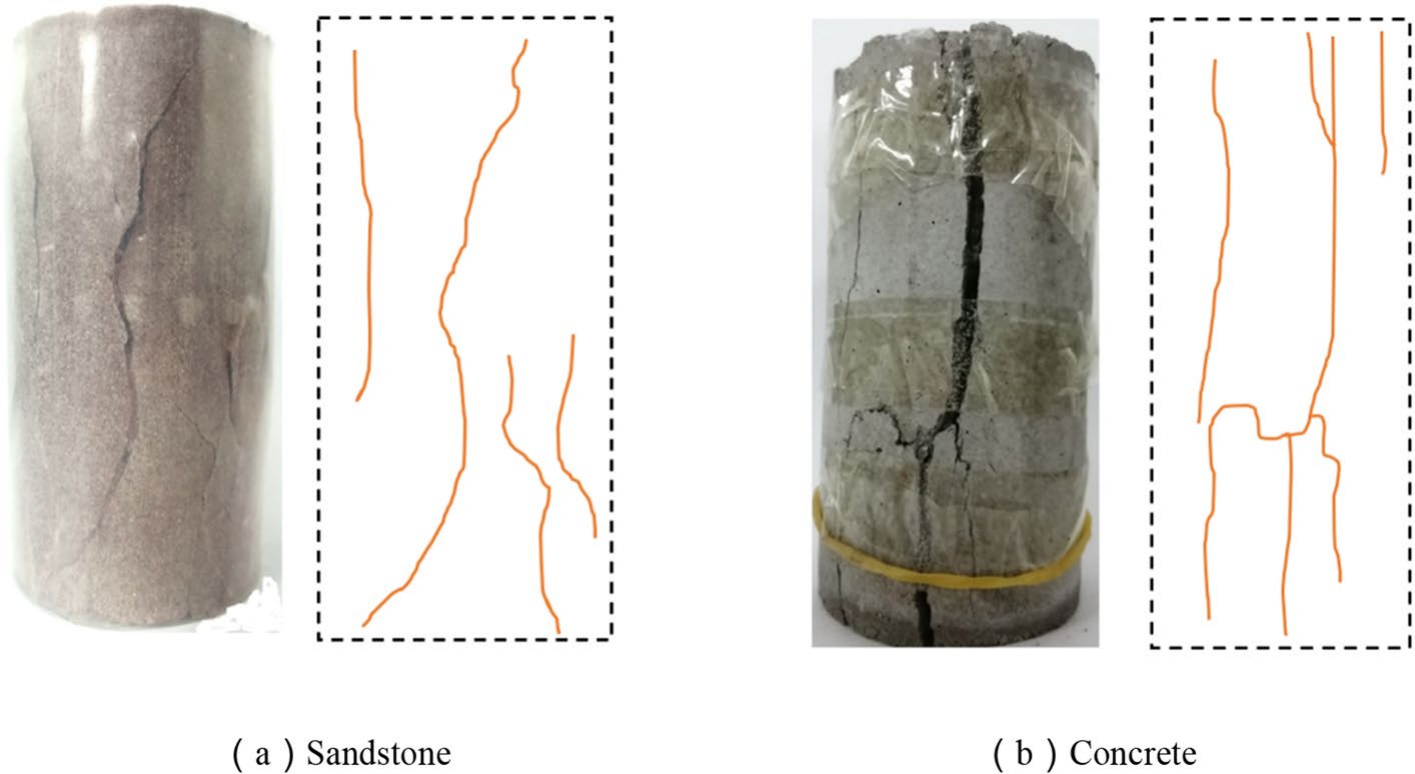
Failure mode of the single material sample.

The failure modes of the rock and concrete are shown in Fig. 3. As shown in Fig. 3a, due to the lack of lateral constraints, brittle failure occurs in sandstone samples, resulting in vertical cracks. Because sandstone is a brittle material, plastic deformation or failure precursor usually does not appear before the failure of sandstone samples. The failure process suddenly occur in the elastic stage, with a sudden drop in stress and a loud sound, which is consistent with the classical failure mode^30^.

As shown in Fig. 3b, cracks first appear at both ends of the concrete sample under the uniaxial compression condition. As the load increases, the crack at the end of the sample expands along the axial direction. When the peak stress is reached, the crack through the sample. Concrete cracks are composed of vertical cracks and inclined cracks, the concrete sample has obvious plastic deformation characteristics during loading.

#### Mechanical properties of a single material

**Figure 4:**
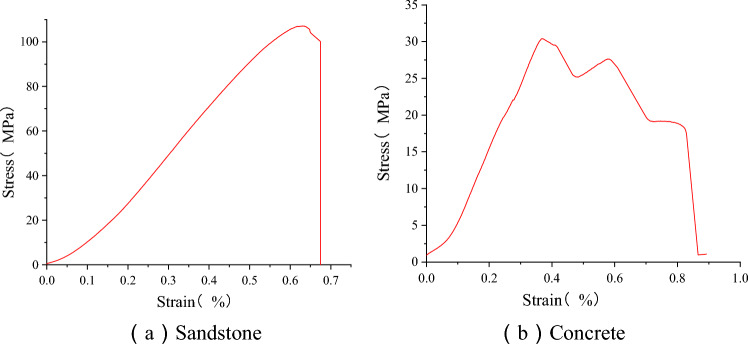
Stress–strain curve of a single material.

The stress–strain data of the concrete and sandstone samples in the uniaxial compression test were collected. The Fig. 4 shows the stress–strain curve of the rock and concrete. The axial strain was monitored in the test. By fitting the linear part of the stress–strain curve, the peak compressive strength and elastic modulus of each sample were obtained, and the obtained partial data were shown in Table 1 below.

**Table 1:** Uniaxial compression test data.

Sample	D/(mm)	H/(mm)	σ_p_/(MPa)	E/(GPa)
Concrete	50.10	100.05	30.39	8.53
Combination	50.02	100.10	39.40	9.35
Sandstone	50.05	100.02	107.12	21.95

As shown in Fig. 4a, there are four stages in the loading process of sandstone, namely the compaction stage, elastic stage, plastic yield stage and fracture failure stage. As sandstone is a brittle material, the plastic stage is not obvious after the compaction and elastic stages. In the elastic stage, the strain increases linearly with the stress. When the stress reaches a peak value of 107.10 MPa, it suddenly decreases to 0 with further application of load, indicating complete failure of the sandstone sample.

As shown in Fig. 4b, according to the stress–strain curve of concrete, the uniaxial compression process can be subdivided into: the compaction stage, elastic linear stage, micro-crack initiation and expansion stage, post-peak failure stage and residual load stage. After the concrete is pressurized, the stress–strain curve has obvious deformation and failure process, and the stress peak value is 30.39 MPa.

### Analysis of test results of sandstone–concrete samples

#### Failure characteristic analysis of sandstone–concrete samples

**Figure 5:**
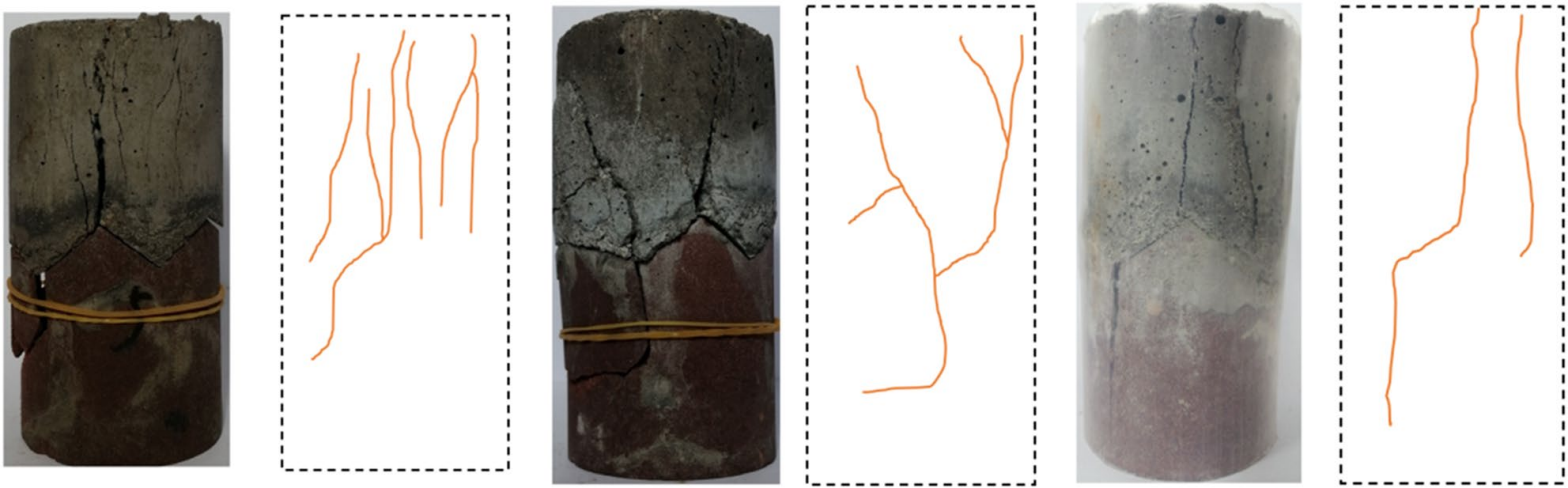
Failure crack diagram of sandstone–concrete combined body.

As shown in Fig. 5, the failure mode of the sandstone–concrete sample is relatively severe and the failure is divided into axial splitting failure and shear failure. There are several cracks throughout the sample, while other cracks are not penetrated. Under the effect of axial pressure, microcracks are first formed at the end, which easily causes stress concentration at the sharp corner of the zigzag triangle interface. Therefore, cracks in the sample are generated and propagated along the sharp corner of the interface. In addition, due to the low strength of the concrete part, the cracks first propagate in the concrete part until the cracks pass through the interface between the two and propagate to the rock part. Eventually, destruction occurs. The failure process reflects the cooperative deformation and failure of the two materials, which is the mechanical failure mode of the continuum.

#### Mechanical properties of sandstone–concrete samples

**Figure 6:**
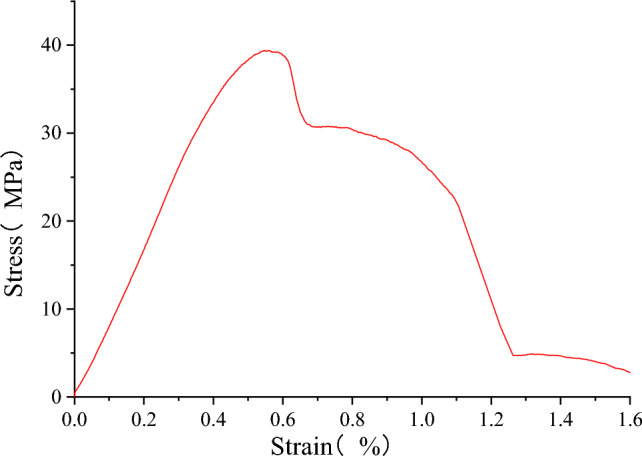
Stress–strain curve of sandstone–concrete combined body.

The stress–strain curves of sandstone–concrete samples are shown in Fig. 6. Through fitting the linear part of the stress–strain curve, the uniaxial compressive strength and elastic modulus of each sample were obtained, and the data obtained were shown in Table 1 below.

The figure shows that the stress–strain curve of sandstone–concrete sample is very similar to that of concrete, showing an obvious plastic failure process. The failure process of the SCCB can be divided into five stages: compaction stage, elastic linear stage, microcrack initiation and propagation process, post-peak failure stage and residual load stage.

The strength of the sandstone–concrete combined body is generally expressed as peak stress (σ_p_). Peak stress refers to the maximum load that sandstone–concrete samples can bear under uniaxial or triaxial compression. It can be seen from the data in Table 1 that the peak stress of the sandstone–concrete composite sample is 39.40 MPa, which is between the uniaxial compressive strength of rock and concrete, and the peak stress of sandstone and concrete is reduced by 63.21% and increased by 29.64%, respectively.

Elastic modulus (E) is an important parameter affecting the deformation of the sandstone–concrete combined body materials. It reflects the ability of the sandstone–concrete sample to resist elastic deformation and the strength of the connection between mineral atoms of the materials.

From Table 1, it is known that the elastic modulus is 9.35 GPa, which is also between the elastic modulus of concrete and sandstone. Compared with sandstone and concrete, the elastic modulus decreased by 57.40% and increased by 9.61%, respectively.

#### Acoustic emission parameter analysis

Acoustic emission (AE) is a nondestructive testing method that can monitor cracks, fractures and other damages of samples under external loads^31–33^. The acoustic emission monitoring system is mainly composed of sensors, preamplifiers and signal acquisition and processing systems. In this study, an acoustic emission monitoring system was used to monitor the uniaxial compression process of the sandstone–concrete sample, to more accurately analyze the failure process of the sample^34–36^.

**Figure 7:**
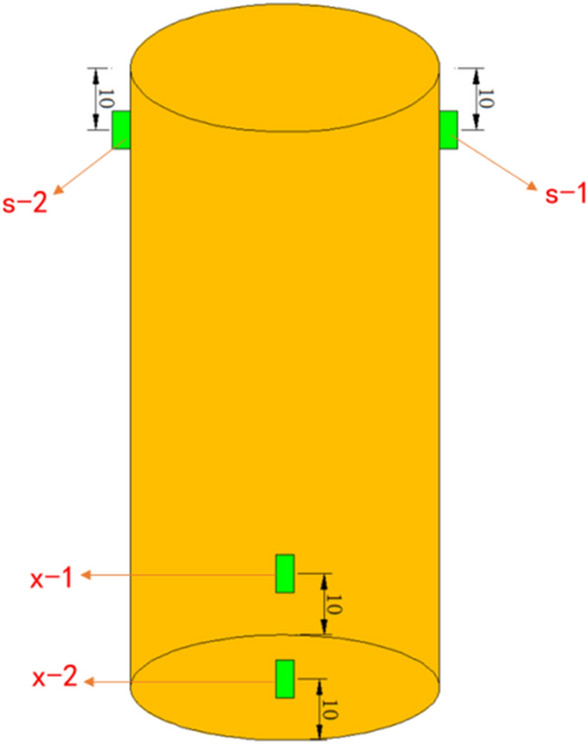
Acoustic emission probe layout.

As shown in Fig. 7, it shows the experimental arrangement of the acoustic emission probe. A total of four acoustic emission probes were arranged in this test, two probes were arranged in the upper part of the sample, the probes were numbered as s-1 and s-2. The probes were arranged at the position of 10 mm from the top of the sample. In the lower part of the sample, two probes, numbered x-1 and x-2, were also arranged at a distance of 10 mm from the bottom of the sample. The lines between the upper probes and the lines of the lower probes were distributed vertically.

Appropriate AE parameters can more accurately reflect the failure process of the sample. Since the variation law of AE parameters is roughly the same in the test process, two AE parameters, namely acoustic emission counting and cumulative acoustic emission counting, were selected. The purpose is to analyze the uniaxial failure process of the sandstone–concrete sample. The internal damage law of the sandstone–concrete sample reflected by the variation law of AE parameters was explored.

**Figure 8:**
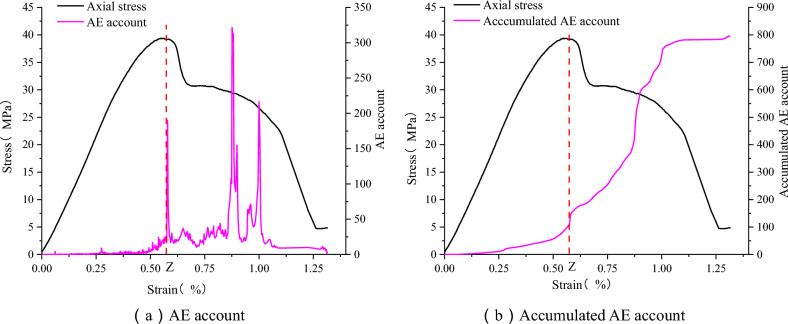
Comparison of acoustic emission parameters and stress–strain curves of the sandstone–concrete sample.

As shown in Fig. 8, the acoustic emission parameters of the sandstone–concrete combined body are plotted against the stress–strain curve. The variation pattern of acoustic emission parameters of the SCCB can effectively reflect the fracture location and fracture process. Meanwhile, the AE counts and cumulative AE counts are less active and remain essentially constant during the compaction stage and the elastic stage, corresponding to the initial loading phase of the stress. This is because at this point the SCCB sample is stressed and there are fewer acoustic emission events from internal microcrack closures. As the strain approaches the Z point, the AE counts begin to fluctuate and the cumulative AE counts begin to show a significant increase. It indicates that at this point the damage within the sandstone–concrete sample intensifies and cracks begin to erupt and develop. After the Z point, the AE counts fluctuate more significantly and the cumulative AE counts increase rapidly. It indicates that at this point the damage within the sample has increased, and the cracks have expanded from the concrete to the sandstone section. Therefore, the sudden increase in the acoustic emission parameter Z, can be used as a precursor to the accelerated damage of the sandstone–concrete sample in the process of studying the failure to the composite material.

## Numerical simulation

### Numerical model establishment and test scheme

FLAC3D is a powerful numerical simulation software for the analysis of mechanical properties of materials, such as subsurface rocks and concrete. To better compare the results of the uniaxial compression test, a numerical model with the same shape and size as the indoor uniaxial test was established.

**Figure 9:**
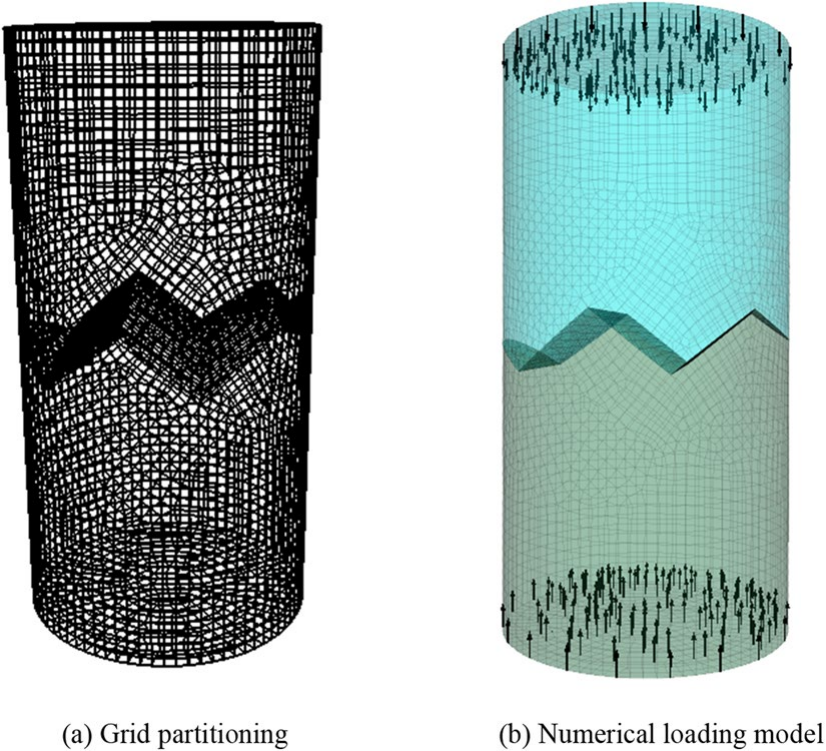
Numerical model.

The process can be divided to following steps for the establishment of the numerical model. Firstly, the grid was divided in Rhino 5.0 software, and the model was divided into upper and lower parts according to the interface form of the sample, and then imported into the FLAC3D software through the external interface to generate the numerical model. Given relevant material parameters in the model, the physical parameters of sandstone and concrete obtained in the test are shown in Table 2 below, and given materials as Mohr–Coulomb constitutive model in FLAC3D software. The model grid is shown in Fig. 9a. The number of units and nodes in the numerical model is 38,527 and 29,681.

**Table 2:** Material physical parameter.

Material	E/(GPa)	C/(MPa)	${{\varphi }}$/(°)	${\sigma }_{{\text{t}}}$/(MPa)
Sandstone	21.95	7.15	36.45	3.0
Concrete	8.53	1.94	50.12	2.2

The boundary conditions of the model are unconstrained in the lateral direction. The forces are applied to the upper and lower surfaces in the axial direction. The loading of the model is controlled by taking a constant displacement loading with a loading rate of 0.1 mm/min, σ_z_ = σ_1_ in the axial direction and σ_2_ = σ_3_ = 0 in the other directions, as shown in Fig. 9b.

### Analysis of numerical simulation results

#### Numerical model stress–strain relationship

**Figure 10:**
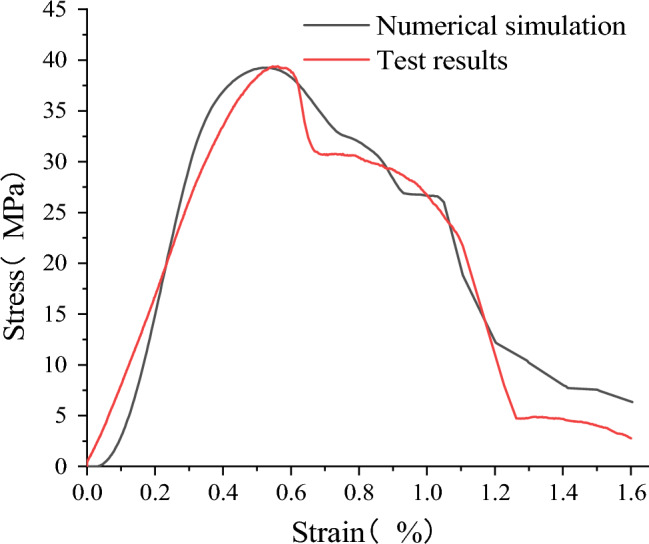
Experimental and numerical simulation stress–strain curves of sandstone–concrete combined body.

The simulation results of the sandstone–concrete combined body in FLAC3D are compared with the stress–strain data obtained from the laboratory uniaxial compression test. As shown in Fig. 10, the variation trend of the simulated curve is consistent with that of the measured curve, and the stage of the loading process is obvious. They are respectively the compaction stage, the elastic stage, the crack initiation stage, the post-peak failure stage and the residual load stage. The value of the peak stress is close to the measured one.

The peak stress obtained by numerical simulation is 39.24 MPa, which is 0.16 MPa different from the measured stress. The elastic modulus obtained by numerical simulation is 11.32 GPa, which is 1.97 GPa different from the measured value. However, the stress peak and elastic modulus obtained by numerical simulation are between sandstone and concrete, which accords with the conclusion of the test. Therefore, the accuracy of the numerical model can be verified.

#### Force analysis of numerical model

**Figure 11:**
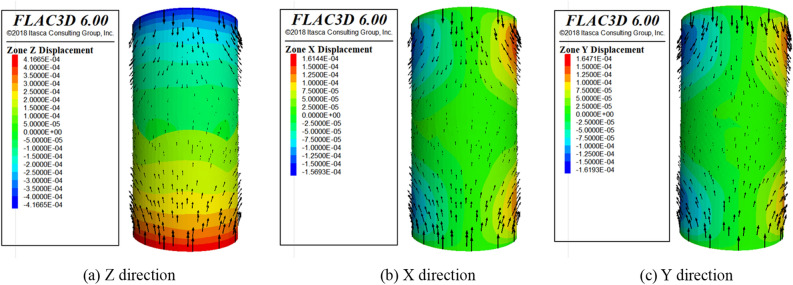
Overall displacement vector diagram.

**Figure 12:**
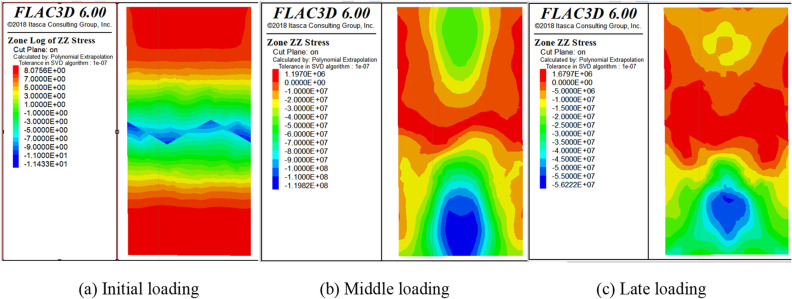
Stress diagram.

**Figure 13:**
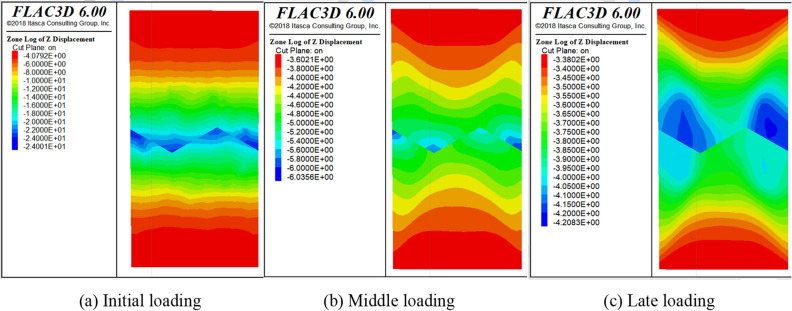
Displacement diagram.

After the reliability of the model is verified by comparing the experimental and numerical stress–strain data. Next, the contour of stress and displacement are analyzed. Displacement and stress monitoring are implemented in the numerical model for the loading process, the contour of displacement and stress are derived at 10 steps intervals. Some representative diagrams of the numerical simulation process are listed in Figs. 11, 12 and 13.

As shown in Fig. 11, the deformation trend of the model can be clearly seen from the following displacement vector plots in X, Y and Z directions. When a load is applied, the SCCB model exhibits sinking deformation at the top and rising deformation at the bottom in the Z-axis direction. The deformation volume is largest at the top and bottom of the model, gradually decreasing towards the middle. As the loading time increases, the high displacement area gradually extends to the central part of the model, demonstrating overall deformation characterized by upper and lower compression and expansion.

As shown in Figs. 12 and 13, the stress and displacement of the numerical model show a height symmetry change with the central axis of the cylinder as the axis of symmetry. Meanwhile, the deformation and failure law of the SCCB sample in the numerical model is known. The deformation at the end of the model is larger than that at the middle part, indicating that the model is destroyed from the end first. In most areas of the upper concrete is tensile stress, so the upper concrete failure occurs first. The shape of the interface leads to stress concentration and a large displacement at the middle part of the model, indicating that cracks and failures occur at the interface. The deformation characteristic of the numerical model is consistent with the experimental results.

#### Mechanical properties of the interface

##### Under uniaxial compression conditions

**Figure 14:**
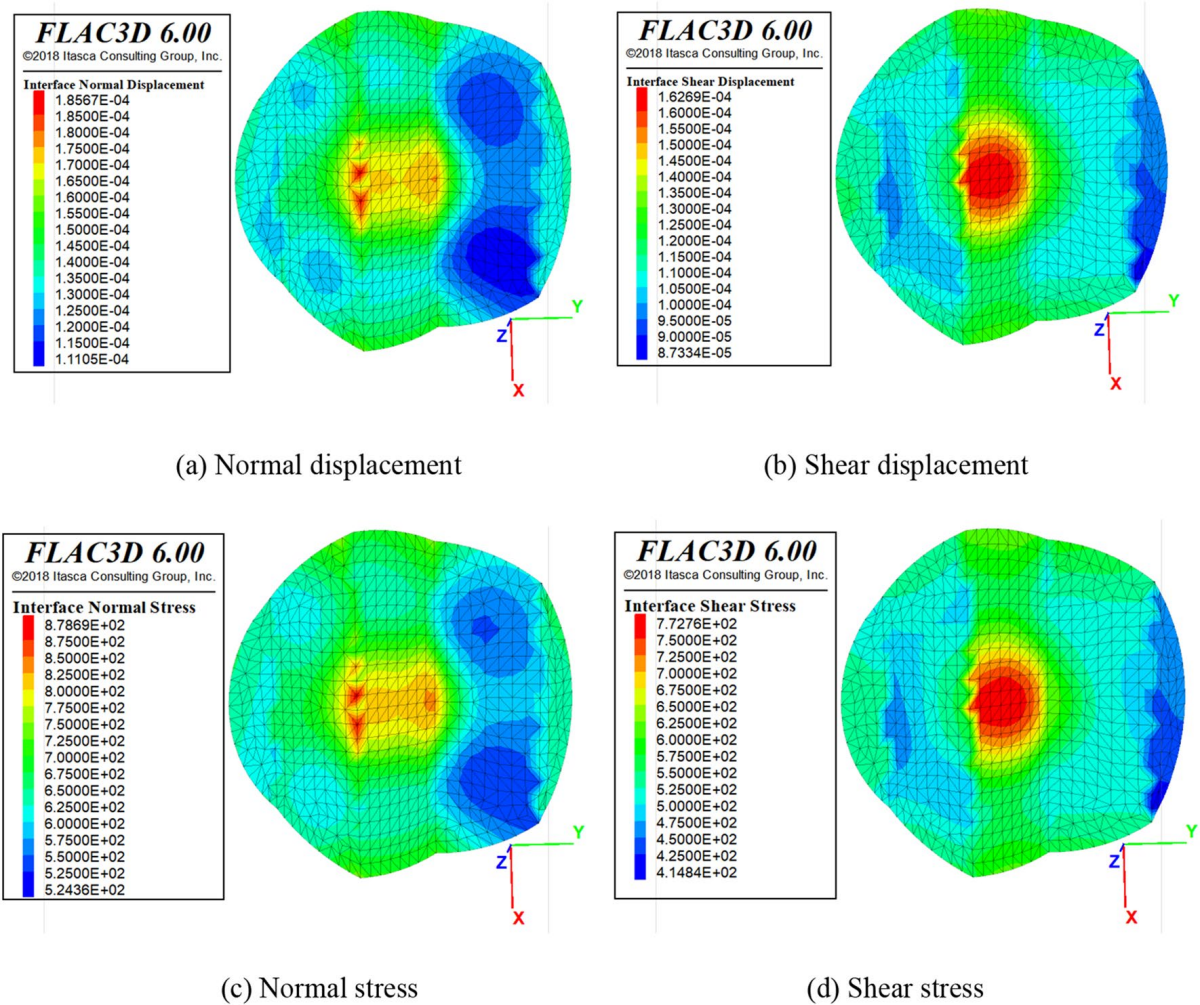
Displacement and stress diagram of the interface.

Since the interface of the sandstone–concrete combined body is serrated, the stress situation of the interface is more complicated. With the help of the interface module of numerical software FLAC3D, the intersection part of the combined body is established and the relevant information of the interface is extracted for research. As shown in Fig. 14, the stress distribution of the interface is not uniform, and there is an obvious stress concentration phenomenon. The analysis of the following normal and tangential stress displacement diagram shows that the larger shear stress is distributed in the central part of the interface. Compared with the edge position, the normal and tangential displacement and stress values generate in the middle part of the interface are larger, and large changes in stress and displacement occur at the interface corners.

Due to the discontinuous interface in the numerical model, the stress and displacement distribution are no uniform under uniaxial compression, especially in the serrated interface area, where stress concentration likely occur. Therefore, the stress concentration coefficient defined in this paper represent the degree of non-uniform stress at the interface under uniaxial compression. By introducing the theory of stress concentration, the peak stress σ_0_ applied to the model at the point furthest from the interface, where there is no stress concentration, is defined as the reference stress. Let $\alpha$ be the ratio of the local maximum principal stress at the interface to the principal stress applied to the model during the compression test, as shown in Eq. (1) below. The stress concentration coefficients in the y and z directions at the position of the center interface being studied.

1 $$\alpha = \frac{{\mathop \sigma \nolimits_{m} }}{{\mathop \sigma \nolimits_{0} }}$$

where σ_m_ is the maximum stress or peak stress at the monitoring point, and σ_0_ is the reference stress.

**Figure 15:**
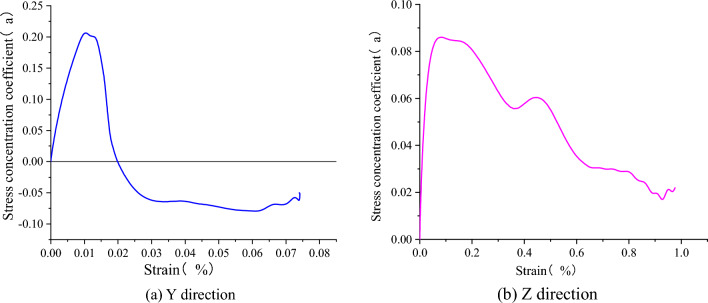
Stress concentration coefficient-strain distribution diagram.

As shown in Fig. 15, the stress concentration coefficient in the y-direction shows that the coefficient increases more rapidly between 0 and 0.01% of the strain rate to reach the peak stress. The strain rate turns negative when it reaches 0.02%, where it changes from tensile stress to compressive stress, indicating that the strain rate reaching 0.02% is the critical point for the change in the stress state of the interface. The change in stress concentration coefficient fluctuates little after a strain rate of 0.03%, indicating that the plastic state is entered at this time.

For the stress concentration coefficient in the z-direction, it is known that the growth rate is faster when the strain rate is 0–0.2%, and this stage is the elastic stage. After 0.2%, the stress concentration coefficient is decreasing and fluctuating, indicating that there is a concentration of stress on the interface and it is entering the plastic stage.

##### Triaxial compression conditions

Based on the above conclusion of the FLAC3D numerical model of uniaxial compression, it is known that the reliability of using FLAC3D to analyze the mechanical failure properties of the SCCB is high.

In conventional triaxial compression tests, constant confining pressure will also exert work on samples, so the numerical simulation of the triaxial compression of the SCCB sample is initiated to simulate the study. The procedure of triaxial loading was defined and run through the fish language in FLAC3D software to simulate the forces in the numerical model with confining pressure of 10, 15 and 20 MPa.

**Figure 16:**
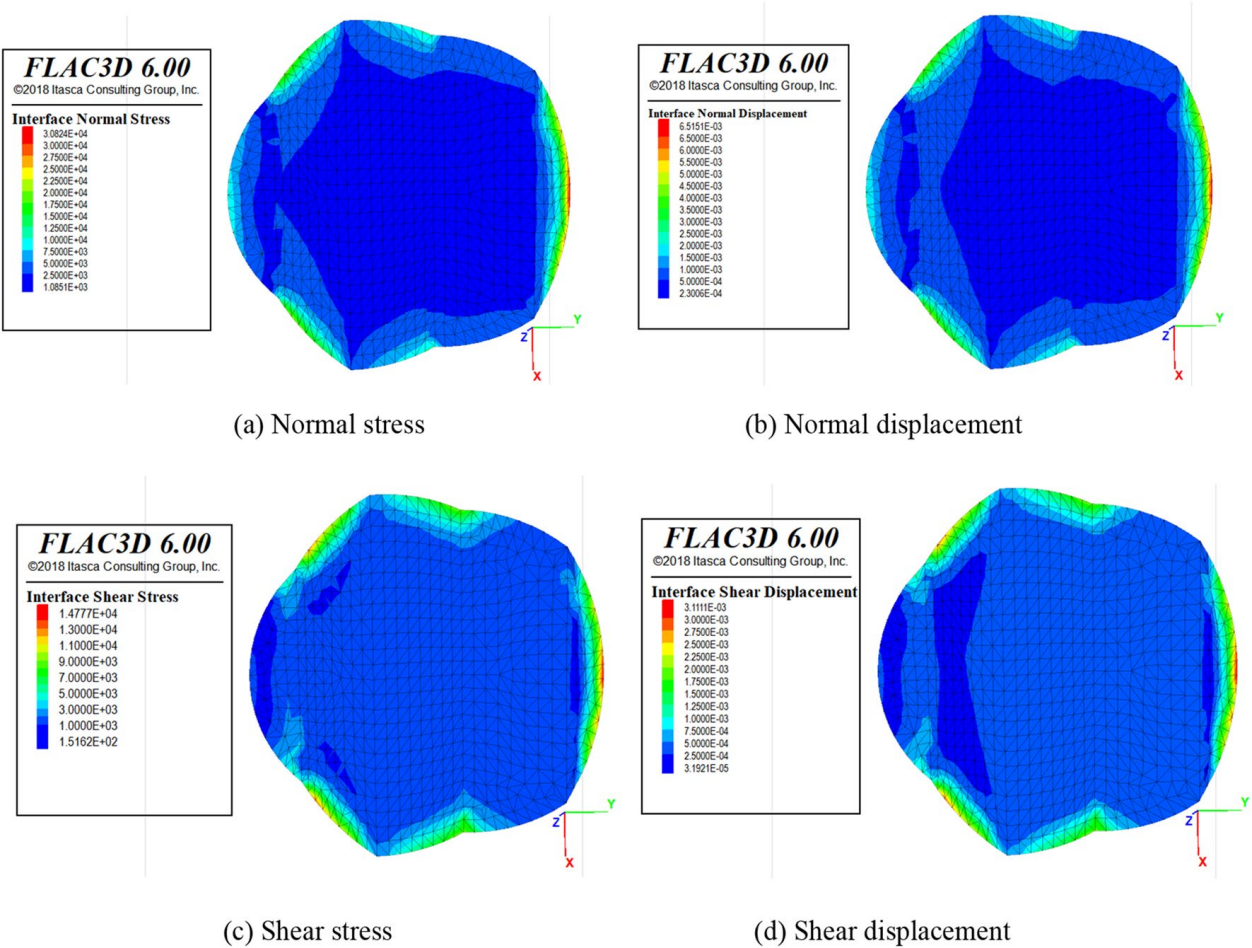
Displacement and stress diagram of 20 MPa confining pressure interface.

Based on the length of the article only the interface stress diagram of the SCCB samples with confining pressure of 20 MPa is presented here. A more uniform distribution of stresses and displacements at the interface is shown in Fig. 16. However, the normal and tangential displacements and stresses at the interface are larger than those in the uniaxial case under triaxial compression conditions. It indicates that the influence of the confining compression has a great influence on the force of the samples.

#### Numerical plasticity zone analysis

**Figure 17:**
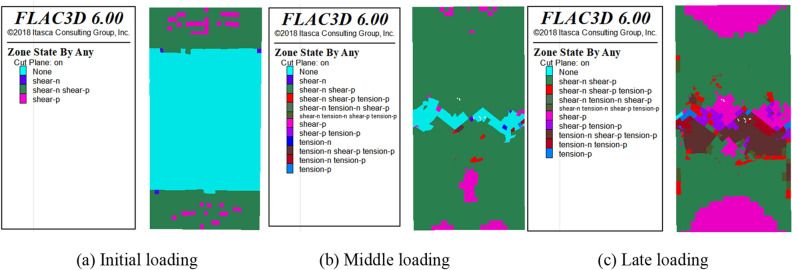
Distribution of the plastic zone of the SCCB under uniaxial compression.

**Figure 18:**
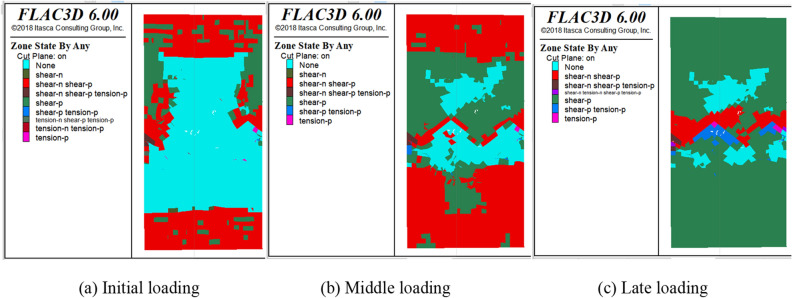
Distribution of the plastic zone of the SCCB at the confining pressure of 20 MPa.

**Figure 19:**
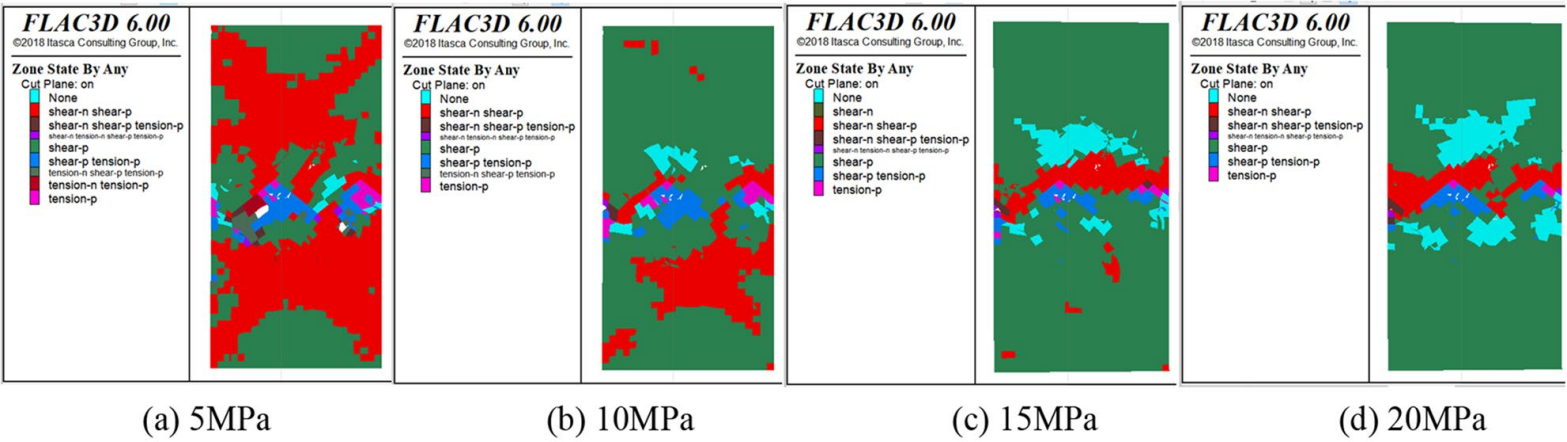
Distribution of the plastic zone of the SCCB under various confining pressures.

The deformation of the plastic zone during model loading was monitored in FLAC3D. The distribution of the plastic zone at more representative time steps was selected for analysis. The distributions of shear failure (shear), tensile failure (tension) and no failure (none) in the plastic zone were counted in FLAC3D, where n and p represent present failure and past failure respectively. The distribution of the plastic zone during uniaxial and triaxial loading of the SCCB is shown in Figs. 17, 18 and 19. Overall, as the time step increases, the plastic zone of the model begins to expand from the end to the middle, and eventually the whole model breaks down.

The plastic zone in the middle of the interface area is combined, with a large number of plastic zones. The main shear failure occurs on the upper part of the interface, while the lower part experiences main tensile failure. During the loading process, changes in the plastic zone can be observed. The upper part of the concrete fails first, followed by the lower part of the rock as the stress increases. The indoor test failure characteristics are used to further verify the accuracy of the test results.

**Figure 20:**
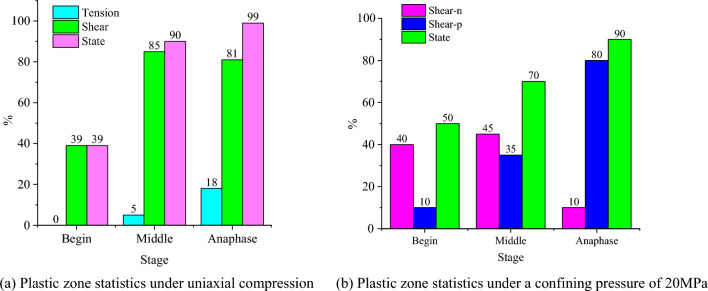
Distribution statistics of plastic zone.

In the case of triaxial loading, under a confining pressure of 20 MPa, there are significantly more undeformed elements in the lower part than in the upper part during the initial loading stage. The plastic zone experiences shear failure, with shear-p representing approximately 40% and shear-n representing about 10%. As the load continues to be applied, the plastic zone gradually expands from the end to the middle of the model and finally runs through the model, with shear-p accounting for approximately 80% and shear-n for about 10%. As shown in Fig. 20, the distribution of the plastic zone under different confining pressures indicates that the plastic zone is primarily associated with shear failure under triaxial compression. The increase in confining pressure leads to a higher proportion of the plastic zone of shear failure in the past when the corresponding number of steps is reached. This indicates that damage will occur in advance under high confining pressure conditions..

### The energy evolution law of uniaxial compression

#### Principle of energy calculation

Quantitative analysis of deformation disruption and damage of the combined body sample is performed using the energy method^37–39^. According to the laws of thermodynamics, there is absorption, storage, release, and transformation of energy in a steady state system. The combined body is essentially a process of energy exchange between the sample and the outside during uniaxial compression. When the sample is loaded, part of the input energy is converted into elastic deformation energy which can recover deformation. Meanwhile, the other part is plastic deformation energy which is generated due to plastic deformation. With the continuous increase of load, the internal damage gradually accumulates to a certain threshold, when the stored energy will be released in the form of kinetic energy, leading to the final failure of the sample. Therefore, the energy method can effectively analyze the deformation failure and damage of the sandstone–concrete samples.

It is assumed that the energy absorbed from the outside by the SCCB sample under the action of external forces is U, and part of the energy is stored as elastic strain energy U^e^, while the rest of the energy is released in the form of dissipative energy U^d^.

2 $$U = U^{e} + U^{d}$$

The total energy U, elastic strain energy U^e^ and dissipation energy U^d^ of the combined body sample in the complex stress state can be expressed in the principal stress space as:

3 $$U = \int_{0}^{{\mathop \varepsilon \nolimits_{1} }} {\mathop \sigma \nolimits_{1} } \mathop {{\text{d}}\varepsilon }\nolimits_{1} + \int_{0}^{{\mathop \varepsilon \nolimits_{2} }} {\mathop \sigma \nolimits_{2} } \mathop {{\text{d}}\varepsilon }\nolimits_{2} + \int_{0}^{{\mathop \varepsilon \nolimits_{3} }} {\mathop \sigma \nolimits_{3} } \mathop {{\text{d}}\varepsilon }\nolimits_{3}$$

where: σ_1_, σ_2_ and σ_3_ are the maximum, intermediate and minimum principal stresses of the sample respectively. ε_1_, ε_2_ and ε_3_ are the principal strains in the three principal stress directions. For uniaxial compression, σ_2_ = σ_3_ = 0, so the total strain energy can be expressed as:

4 $$U = \int_{0}^{{\mathop \varepsilon \nolimits_{1} }} {\mathop \sigma \nolimits_{1} } \mathop {{\text{d}}\varepsilon }\nolimits_{1}$$

5 $$U^{e} { = }\frac{1}{{2E_{i} }}[\mathop \sigma \nolimits_{1}^{2} + 2\mathop \sigma \nolimits_{3}^{2} - 2\mu (\mathop \sigma \nolimits_{3}^{2} + 2\mathop \sigma \nolimits_{1} \mathop \sigma \nolimits_{3} )] \approx \frac{1}{{2E_{0} }}\mathop \sigma \nolimits_{1}^{2}$$

6 $$U^{d} = U - U^{e}$$

where E_i_ is the elastic modulus of the sample at the corresponding moment, µ is the Poisson's ratio of the sample, and E_0_ is the elastic modulus of the sample.

#### Energy evolution analysis

According to the above energy calculation Eqs. (2)–(6), the input total energy density, elastic energy density and dissipated energy density of the numerical and test the combined body under uniaxial compression can be obtained.

**Figure 21:**
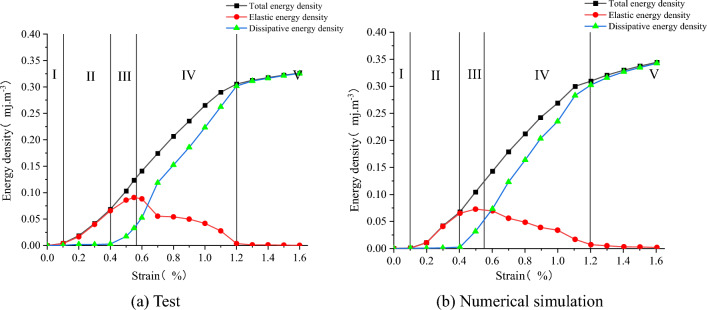
Energy evolution law in uniaxial compression failure process.

As shown in Fig. 21, the curve variation of the energy of each part of the test is similar to the value. Combined with the stress–strain relationship analysis, the energy change process can be segmented. By analyzing Fig. 21, the energy evolution law of the SCCB samples under uniaxial compression can be obtained as follows:

1. During the compression–density stage (I), the strain energy of all parts of the sample increases gradually with the increase of axial strain. The increase in releasable elastic strain energy is greater than that of dissipative strain energy, indicating that the energy input from the outside is primarily converted into releasable elastic strain energy for storage. However, the closure of primary micro-defects in the sample also consumes a certain amount of strain energy.
2. In the online elastic stage (II), the energy absorbed from the external source is primarily utilized for the elastic deformation of the internal load-bearing structure of the sample. It is predominantly converted into releasable elastic strain energy storage, while the dissipated strain energy remains nearly unchanged or increases slightly.
3. In the crack emergence stage (III), external energy continues to be input to the sample, primarily stored as releasable strain energy. However, the dissipated strain energy increases significantly due to a large number of new fine cracks in the sample, and the proportion of plastic deformation in the total deformation also increases significantly. Irreversible plastic deformation occurs in the sample, and most of the absorbed energy is converted into dissipative energy. This promotes the sprouting, expansion, and penetration of microcracks, leading to a rapid increase in the density of dissipative energy.
4. In the post-peak damage stage (IV), after the axial stress reaches its peak strength, the sample can still absorb energy from the outside. However, the rate of total strain energy increase tends to slow down slightly, while the dissipated strain energy increases rapidly. Plastic deformation and macro crack penetration dissipate a significant amount of energy, causing the strength of the sample to gradually decrease. Simultaneously, a significant amount of stored elastic strain energy can be rapidly released in the form of kinetic energy, surface energy, frictional heat energy, etc., leading to the overall instability and failure of the sample.
5. In the residual load stage (V), the dissipated energy density gradually approaches the total energy density, while the elastic energy density gradually decreases to nearly zero until the sample completely fails and loses its load-bearing capacity.

This indicates that the process of uniaxial compression of the sample involves energy conversion. The cracks inside the sample will undergo unstable expansion, connectivity, and frictional slip, all of which require a significant amount of energy consumption. Consequently, the sample's failure can be assessed by the change in energy density.

#### Linear energy storage law of sandstone–concrete sample

**Figure 22:**
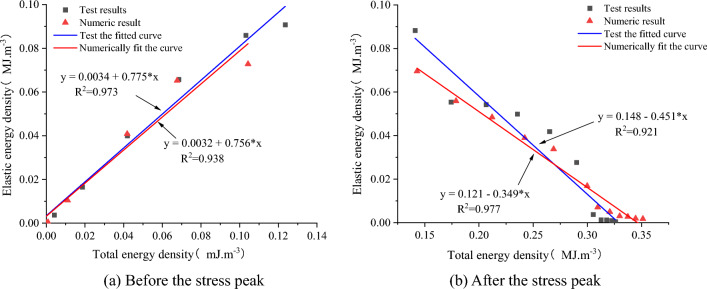
Graph of elastic energy density versus total input energy density.

The relationship between the elastic energy density and the total input energy density before and after the peak stress is plotted, as shown in Fig. 22. The elastic energy density U^e^ increases with the increase of the total input energy density U before the peak. The elastic energy density U^e^ decreases with the increase of the total input energy density U after the peak, both showing an obvious linear relationship. Through linear fitting of each data point, the fitting function expression between the elastic energy density of the sample and the total input energy density was obtained.

The results of the fitting function show that the relationship between the elastic energy density and the total input energy density also accords with the linear function $U^{e} = aU + b$. The effect of the small intercept b value on the functional relationship can be ignored. The function is approximately in the form of $U^{e} = aU$. It can be considered that the ratio between the elastic energy density of the SCCB and the total input energy density is a constant value, and this ratio $a$ is defined as the rock compression energy storage coefficient. The energy storage coefficient of compression reflects the storage capacity of elastic energy of the sandstone–concrete sample. The larger the energy storage coefficient, the greater the ability to store elastic energy. The $a$ before the stress peak is 0.756, 0.775, so it shows that the energy storage capacity of the combined body before the stress peak is strong. The $a$ after the stress peak is − 0.349, − 0.451, respectively, indicating that the energy storage capacity of the combined body is weak after the stress peak, which is the stage of releasing elastic energy.

#### Energy damage evolution law of sandstone–concrete sample

The loading process of the model is accompanied by energy changes, mainly in the form of energy accumulation, dissipation and release^40–43^. The accumulation of dissipation energy of the sample can cause damage to the material of the combined body. The leaded deterioration of the material is the main cause of the failure of the SCCB sample. Therefore, the damage stage of the model can be distinguished according to the change characteristics of the dissipated energy.

Therefore, this paper proposes a damage parameter D that quantifies the energy damage of the SCCB sample, and defines the ratio of dissipated energy $U^{d}$ during the process and dissipated energy $U_{\max }^{d}$ during destruction as the damage factor $\beta$:

7 $$\beta = \frac{{U^{d} }}{{U_{\max }^{d} }}$$

8 $$D = \left( {1 - \frac{{\sigma_{{\text{r}}} }}{{\sigma_{{\text{c}}} }}} \right)\frac{{U^{d} }}{{U_{\max }^{d} }}$$

where σ_r_ is the residual strength.

**Figure 23:**
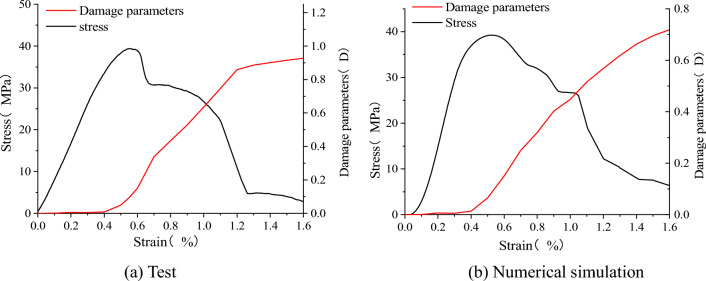
Damage evolution during uniaxial compression.

As shown in Fig. 23, the damage evolution law of the sandstone–concrete sample under uniaxial compression was determined. The variation trend of the damage curves obtained from experiments and numerical results is consistent, indicating a nonlinear change process. In the elastic stage, the damage parameter D is equal to 0. As the load continues to be applied, the axial strain gradually increases, cracks gradually occur in the sample, and damage accumulates over time. When the strain reaches approximately 0.4%, the damage parameter starts to increase rapidly, indicating the propagation of cracks. At a later stage, the growth rate of the damage curve slows down, and when the damage accumulation reaches a certain threshold, the sample will be destroyed. Overall, the change in the damage curve is gradual, indicating that the damage process of the sandstone–concrete sample exhibits plastic behavior. Comparison of the cumulative AE count curves in Fig. 8 reveals that the damage curves and the cumulative AE counts exhibit similar variations, suggesting that the damage curves, akin to the acoustic emission parameter curves, can effectively depict the damage during uniaxial loading of the SCCB.

### Numerical triaxial compression energy evolution law

#### Numerical energy evolution law

**Figure 24:**
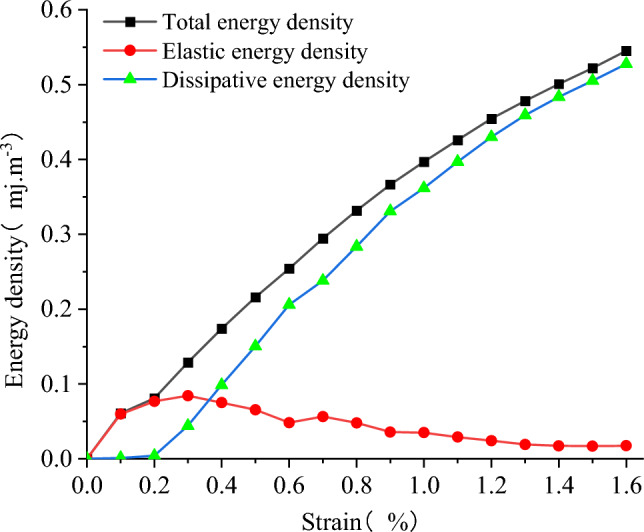
The energy changes in the deformation process when the confining pressure is 5 MPa.

The stress–strain values of the model under triaxial compression can be derived from FLAC3D, and the input total energy density, elastic energy density and dissipated energy density of the model under triaxial compression can be calculated by the above Eqs. (2)–(6). As shown in Fig. 24, the variation process of strain energy of each part of the model with axial strain when confining pressure is 5 MPa. By comparing Figs. 21 and 24, the variation law of strain energy of each part of the model under conventional triaxial compression and uniaxial compression is basically the same. It can be divided into five stages to analyze the process of triaxial compression, which will not be detailed here. Where, U and U^d^ increase with the increase of strain, and the variation trend of U^e^ has the same stage characteristics as that under uniaxial conditions, as mentioned Shen and Zhao et al.^44–48^. Compared with the energy characteristics of the model under uniaxial compression, the total strain energy absorbed, the dissipated strain energy and the releasable strain energy of the model under conventional triaxial compression are larger than the energy values of the corresponding state model in uniaxial compression.

**Figure 25:**
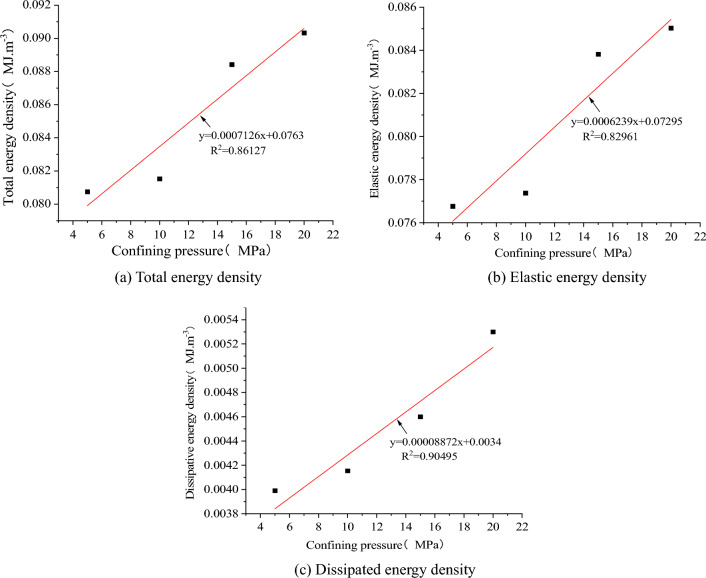
The relationship between energy and confining pressure at stress peaks.

**Figure 26:**
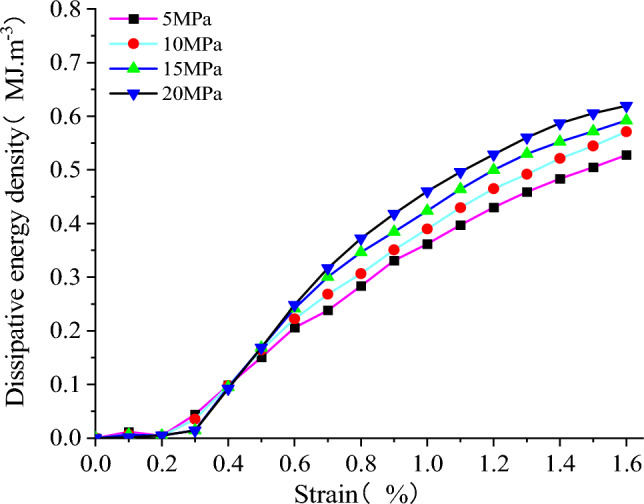
Relationship between dissipated energy density and strain.

**Figure 27:**
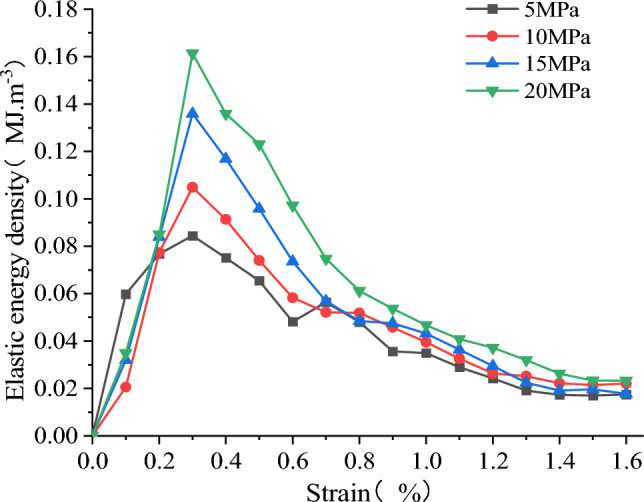
Relationship between elastic energy density and strain.

As shown in Fig. 25, the strain energy of each component at the peak stress of the model increases with the rise in confining pressure. It exhibits a strong linear relationship with the confining pressure. The linear regression equations exhibit a high correlation. As shown in Fig. 26, the energy density dissipation of the model increases with higher strain, and the dissipated energy also increases with the rise in confining pressure. The dissipative energy density at high confining pressure is greater than that at low confining pressure, and its peaks are 0.528, 0.571, 0.592, and 0.619 MJ/m^3^, respectively. As shown in Fig. 27, the peak elastic energy density of the model increases with the rise in the confining pressure. The peaks are 0.084, 0.105, 0.136, and 0.161 MJ/m^3^, respectively. The overall trend of variation initially increases and then decreases with the strain.

**Figure 28:**
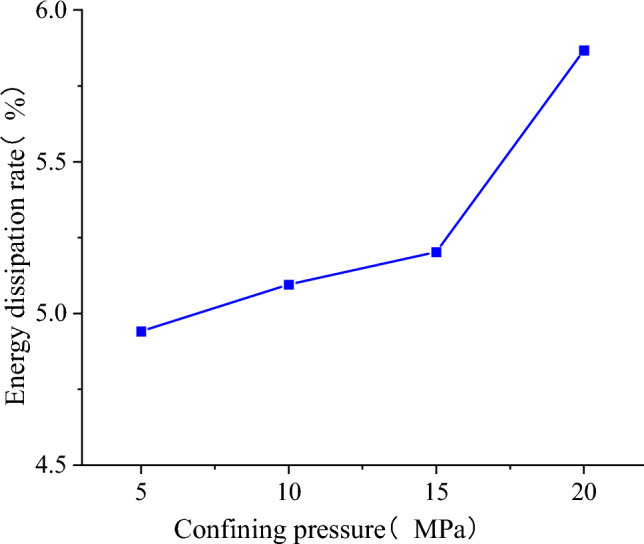
Energy dissipation rate at the peak point.

The energy dissipation rate is defined as the ratio of the dissipated energy to the total energy. As shown in Fig. 28, the energy dissipation rate at the peak increases with the rise in confining pressure. The model's energy dissipation is inevitably linked to damage development, suggesting that the damage of the SCCB sample intensifies gradually during the pre-peak stage as the envelope pressure increases.

#### Triaxial compression damage evolution law

Based on the definition of the damage parameters in "Energy damage evolution law of sandstone–concrete sample" and the resulting damage evolution law, damage curves were obtained for the conventional triaxial compression process of the model at different confining pressures. The compressive strength, residual strength and dissipation energy at damage of the samples at different confining pressures are shown in Table 3 below.

**Table 3:** σ_c_, σ_r_ and U^d^_max_ of composite samples under different confining pressures.

σ_3_/MPa	σ_r_/MPa	σ_c_/MPa	U^d^_max_
5	21.83	49.60	0.505
10	25.03	55.53	0.545
15	22.25	63.43	0.577
20	15.79	68.49	0.614

**Figure 29:**
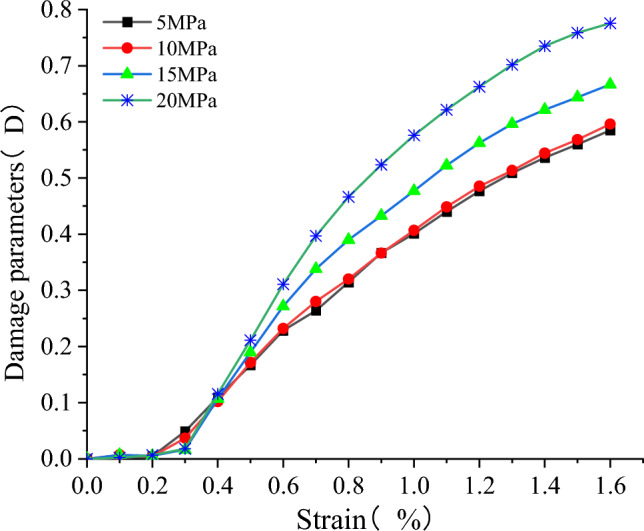
Damage evolution law of samples during deformation and failure under different confining pressures.

According to Formula (8) and Table 3, the damage evolution curves of the model under triaxial compression loading were obtained, as shown in Fig. 29. The figure shows that the damage changes little and is 0 in the compaction stage and elastic stage, and the damage increases with the increase of axial strain in the later stage. The larger the confining pressure is, the larger the damage variable is. With the increase of confining pressure, the damage parameters were 0.585, 0.596, 0.666 and 0.776, respectively. Similar to the damage curve for the uniaxial compression process, the damage curve for the combined body model grows slowly during deformation damage in triaxial compression conditions, showing a smooth failure process.

## Conclusion

In this paper, the mechanical properties and failure modes of the SCCB samples are investigated by a combination of uniaxial compression tests and numerical simulations. The following conclusions are obtained:

1. The deformation and failure characteristics of the SCCB sample are similar to those of traditional concrete. As the load increases, the failure process can be divided into five stages. First, the upper concrete and the interface are damaged, and the overall deformation and failure of the two materials are coordinated. The main failure mechanisms are splitting and shear. The peak stress and elastic modulus fall between those of sandstone and concrete.
2. The internal damage and crack growth of the SCCB sample can be assessed based on the acoustic emission count and the fluctuation in cumulative acoustic emission count. Under uniaxial compression, the shape of the interface leads to stress concentration. The study reveals that the shape of the interface has a significant impact on the stress in the y-direction, causing a transition from tensile stress to compressive stress. In triaxial compression, the confining pressure significantly affects the stress at the interface, resulting in a more uniform stress and displacement distribution compared to uniaxial compression.
3. Under uniaxial compression, the energy curves of each section of SCCB exhibit similarities to those observed in experiments and numerical simulations, and can be categorized into five stages. Energy is stored before reaching peak stress, and then released after reaching peak stress. Under the triaxial compression numerical simulation, the energy of each component is significantly higher than that under uniaxial compression conditions. It shows a positive linear correlation with the confining pressure, and the energy dissipation rate increases as the confining pressure rises.
4. Under uniaxial compression, the damage curve of the SCCB indicates a gradual increase in damage with strain, characterized by plastic failure. Under triaxial compression numerical simulation, the damage evolution of the SCCB is similar to that under uniaxial compression, but it increases with the rise in confining pressure. Additionally, the plastic failure process is also observed.

## Data Availability

Some or all data, models, or codes that support the findings of this study are available from the corresponding author (Zienshen@126.com) upon reasonable request.
